# Structural disadvantage and HIV risk – comparing risk factors between trans women’s partnerships with cis men and trans women sexual partners

**DOI:** 10.21203/rs.3.rs-4492723/v1

**Published:** 2024-06-13

**Authors:** Erin C. Wilson, Bow Suprasert, Dillon Trujillo, Sofia Sicro, Christopher J. Hernandez, Caitlin M. Turner, Willi McFarland, Sean Arayasirikul

**Affiliations:** San Francisco Department of Public Health; San Francisco Department of Public Health; San Francisco Department of Public Health; San Francisco Department of Public Health; University of California, Los Angeles; University of California, San Francisco; San Francisco Department of Public Health; University of California Irvine

**Keywords:** Trans women, Sexual partners, HIV risk, Structural disadvantage, Stigma, Social determinants

## Abstract

**Introduction:**

Little is known about differences in HIV risk for trans women by partner gender, particularly with respect to social determinants and partner-level circumstances that affect behavior. We examined differences in demographic, social determinants, and HIV-related risk behaviors for trans women with cis men and trans women sexual partners.

**Materials and Methods:**

Data are from a cross-sectional survey of trans women and their sexual partners conducted between April 2020 and January 2021. Interviews were held remotely during shelter-in-place due to Covid-19 via videoconference. Analysis characterizedassociations between HIV risk and protective behaviors comparing trans women with cisgender men partners to trans women with non-cisgender sexual partners.

**Results:**

A total of 336 sexual partners were identified from 156 trans women. Trans women with cis men partners had significantly less education and employment and more incarceration and recidivism than trans women with trans women partners. Trans women and their cisgender men partners had shared experiences of unstable housing, incarceration, and HIV. Trans women with cisgender men partners reported significantly more sex exchange partners, receptive condomless sex, receptive or insertive condomless sex while using substances, and HIV infection compared to trans women with trans women partners.

**Conclusions:**

Trans women with cisgender men sexual partners faced higher HIV risk than trans women with trans women sexual partners. These risks may be related to the social and economic drivers that both trans women and their cis men partners faced, including barriers to education and employment, along with incarceration and recidivism. Interventions focused on economic stability, workforce development and post incarceration re-entry support for housing and employment for trans women with cis men partners and the cisgender men partners as well may have the most impact on reducing HIV risk and incidence.

## Introduction

Trans women have disproportionate burden of HIV throughout the world, yet the proximal cause of HIV acquisition is largely unknown. Although links between large structural factors like anti-trans discrimination and HIV risk are well demonstrated, less is known about the impact of structural drivers on the contexts and behaviors of trans women and their sexual partners (1, 2).

To date, little research has been conducted to examine the risk environment for trans women and their sexual partners. A systematic review of cisgender men (hereafter, cis men) sexual partners of trans women estimated an HIV prevalence of 30.6%, with almost half (46.1%) engaging in condomless anal sex with trans women (3). A recent online study of cis men who have sex with trans women found that cis men who identify as heterosexual were more likely to report exchange sex, briefer relationships, sex outside partnerships, and less pre-exposure prophylaxis (PrEP) use than cis men with other sexual identities (4). Our prior study found that African American and Latina trans women report having sexual partners of the same race, which gives some insight into their sexual networks (5). Other studies have shown that trans women are most likely to engage in sexual risk behavior with cis men partners and primary partners (2, 6, 7). Research with other populations suggests that condomless sex is more common in primary partnerships due to age and income discrepancies (8), relationship length and seriousness (9), and intimacy and trust (10). In addition, trans women may have power imbalances in relationships due to stigma and unmet basic needs, which in turn may result in sexual risk behavior (11). Yet, few data exist to explain why and how patterns in partnering result in HIV risk. Social determinants are particularly relevant for trans women due to their multiple stigmatized identities, which their sexual partners may also experience (12). Stigma may impact access to PrEP and HIV care for both trans women and their sexual partners, which further impacts risk within sexual networks (13). Factors such as mass incarceration, residential segregation, and socio-economic factors may explain individual risk and risk within sexual partnerships, which also affects HIV transmission, as found in other populations (14).

Data are needed to focus HIV prevention efforts with trans women, understand the context of HIV risk within different partnership types and contribute information on the sexual partners of trans women. The present study was conducted to determine if trans women with cis men partners exhibit more structural disadvantage and engage in more sexual risk behavior than when partnered with trans women. We also examined differences in demographic, behavioral and social determinants for trans women and partners. We tested the hypothesis that social determinants and HIV risk and protective behaviors were significantly worse for trans women with cis men partners. We also provide data on the social determinants of partners.

## Methods

Data for this analysis were collected from survey data collected from April 2020 to January 2021. Surveys were interviewer-administered via Zoom during shelter-in-place ordinances due to the Covid-19 pandemic. Trans women were also recruited through word-of-mouth, social media advertisements, and outreach on dating apps. Participants received $50 for participating in the survey, and $20 for referring additional participants. Trans women were eligible for the study if they were 18 years of age or older, identified as a trans woman, lived in California, and spoke English or Spanish.

### Measures

Trans women participants were asked demographics questions like gender identity, sexual orientation, race/ethnicity, and social determinants like education, employment, age, incarceration, housing stability, income, health insurance, and HIV status of themselves and their sexual partners. Responses of don’t know, refuse to answer, and not applicable to any of the demographic questions were coded as missing. Data on sexual partners reported by trans women relied on their knowledge of up to their 3 most recent partners in the last 12 months. The number of sexual partners was assessed by “How many people did you have vaginal or anal sex with in the past 12 months?”. Participants were also asked “In the past 12 months, what types of sexual partners have you had vaginal or anal sex with?”. Options were (1) main, (2) causal, and (3) exchange. Two additional questions were also asked to assess (1) number of exchanged partners and (2) whether trans women exchanged sex for drugs or money in the past 3 months.

HIV risk and preventive behaviors were assessed via self-report. We assessed insertive condomless anal sex, sex receptive condomless anal sex, and insertive and receptive condomless anal sex while using substances in the past 12 months. Responses of don’t know and refuse to answer were coded as missing. Concurrent sex was assessed by “During the time you were having a sexual relationship with your sexual partners, did you have sex with other people?” (Yes/No) and “As far as you know, during the time you were having a sexual relationship with your partners, did they have sex with other people?”. Options to the second question were (1) Definitely did not, (2) Probably did not, (3) Probably did, and (4) Definitely did. Answering yes to the first question and answering “probably did” (3) or “definitely did” (4) to the second question were coded as having concurrent sex. Injection drug use was assessed by asking “Have you injected substances in the past 6 months?”.

History of HIV testing was asked as “Have you ever been tested for HIV?”. Participants were asked if they were living with HIV or not. PrEP use ever and in the last six months was assessed. For those who responded that they were living with HIV, engagement in HIV care was assessed with questions on initial linkage, current healthcare engagement, and whether they were taking their HIV medications. Viral load was assessed with the question, “Was your most recent viral load detectable or undetectable?”. HIV status of the participants’ partner was asked as “To the best of your knowledge what is your sexual partner’s HIV status?”. Participants were also asked “How do you know their HIV status?”. Options were (1) They told me, (2) They showed me their HIV test results, (3) We got tested together, and (4) Other. Only responses 2 and 3 were used for the analysis. Response of don’t know, refuse, and not applicable were coded as missing.

### Data Analysis

We tested the hypothesis that risk behaviors were most frequent with cis men partners (6, 7) compared to partners who were trans women. We also assessed whether mixing between high and low risk individuals or drug use within a racial network explain disparities in HIV risk among trans women and their sexual partners.

The partnership-level data in this study is egocentric and relied solely on trans women’s perception and knowledge of their partners. For this paper, only three most recent partners were used to create an aggregate of trans women partnership data and to classify trans women into two groups. Gender was asked as “What is your partner’s gender?”. Options were (1) cis man and (2) transgender women. Responses of don’t know, refused to answer, or not applicable were coded as missing and removed from further analyses. Gender of their three most recent partners were used to classify trans women into trans women with cis men partners. Specifically, if at least one of their three most recent sexual partners is a cis man, they were categorized as a trans woman who has sex with cis men. Trans women were categorized as a trans woman with non-cis men partners if none of their three most recent partners is a cis man. These categories were also used to create variables such as exchanged sex for drugs or money in the past 3 months, insertive condomless anal sex, receptive condomless anal sex, sex while using substance, etc. For instance, if a trans woman participant answered that they had insertive condomless anal sex with at least one of their three most recent partners, their responses were classified as yes.

Chi-squared tests were used to assess differences in characteristics and HIV risk and protective behaviors between trans women with and without cis men partners. Fisher’s exact tests were used in the case that cell count fell below 5. Student’s t-tests were also conducted for continuous variables such as number of exchanged partners in the past 12 months. Basic demographic of trans women and their three most recent cis men partners were used to identify concordance in socio-demographic factors within pairs of trans women and cis men. Bivariate and multivariate logistic regressions were conducted to assess direction and strength of association between HIV risk and protective behaviors comparing trans women with to without cis men partners. Exact logistic regression was also conducted in the case of small sample size such as HIV positive trans women. A significant level was set at p < 0.05. Participants provided signed informed consent to participate. The study was approved by the Institutional Review Board (IRB) of the University of California, San Francisco (IRB No. 18–26447).

## Results

Table 1 describes characteristics of trans women and up to three of each trans woman’s sexual partners. A total of 336 sexual partners were described by 156 trans women. Most partners were identified by trans women as being assigned male sex at birth (97.6), cis men (81.9%), straight (59.1%), employed (65.5%), between the ages of 18–39 years old (69.9%), never incarcerated (68.5%), stably housed (75.3%), and HIV negative (89.7%). Sexual partners were identified as mostly white race/ethnicity (42.0%), followed by African American (23.7%) and Latino/a/x (21.3%). The majority of trans women identified as straight (47.1%), employed (48.1%), between ages 18–39 years old (66.0%), have been incarcerated (53.3%), stably housed (66.2%), and HIV negative (79.5%). Trans women were mostly white (27.6%), multi-racial/ethnic (25%), and Latino/a/x (23.7%).

Table 2 presents data comparing characteristics of trans women in partnerships with cis men to trans women with non-cis men sexual partners. Of 156 trans women, 130 (85.5%) were classified as having at least one cis man sexual partner, while 22 (14.5%) were classified as having non-cis men partners. All trans woman classified as having non-cis man partners identified their partners as trans women (N = 22). Of 130 trans women with cis men partners, more than half identified partners as main (68.5%) and casual (63.0%). One-fourth of trans women with cis men partners identified their partners as exchange partners (25.0%), while only one trans woman with trans women partners had an exchange partner (5.6%). Among 130 trans women with cis men partners, slightly more than half identified as straight/heterosexual (55.0%). No trans women with trans women partners identified as straight/heterosexual and many identified as queer (31.8%), pansexual (27.3%), and gay or lesbian (18.2%). Out of 130 trans women with cis men partners, many identified as Latino/a/x (27.7%) followed by multiracial/ethnic (24.6%), White (21.5%), and African American/Black (16.9%). Most trans women with trans women partners identified as White (63.6%). While few trans women in our study had vaginoplasty (N = 18, 11.8%), most with vaginoplasty had cis men sexual partners (N = 14, 77.8%).

Significantly more trans women with trans women sexual partners had a college degree compared to trans women with cis men partners (50% vs. 27.7%, p = 0.036). Nearly half of trans women with cis men partners reported being employed (45.4%), while significantly more trans women with trans women partners reported being employed (72.7%, p = 0.018). Among 59 employed trans women with cis men partners, 5 (8.5%) reported having at least one unemployed partner. Of 16 employed trans women with trans women partners, 1 (6.3%) reported having at least one employed partner. Among 44 unemployed trans women with cis men partners, 25 (56.8%) reported having at least one employed partner. Two of 4 (50.0%) unemployed trans women with trans women partners reported having at least one employed partner. More than half of trans women with cis men partners reported a history of incarceration (57.8%) compared to 18.2% of trans women with trans women partners (p < 0.01). Trans women with cis men partners were incarcerated 3.9 more times than trans women with trans women partners [4.1 (SD = 7.5) vs. 0.2 (SD = 0.4), p = 0.02]. Most trans women with cis men partners reported having stable housing (63.9% vs. 81.8%, respectively). Although the difference was not significant, many more trans women with cis men partners reported unstable housing than trans women with trans women partners (36.2% vs. 18.2%). Among 83 trans women with cis men partners with stable housing, 10 (12.0%) of them reported having at least one partner who was not stably housed. Of 18 trans women with trans women partners with stable housing, 1 (5.6%) reported having at least one partner who was not stably housed. Among 47 trans women with cis men partners who reported unstable housing, 8 (17.0%) reported having at least one partner who had stable housing. All trans women with trans women partners who were unstably housed reported having at least one partner who had stable housing.

Table 3 shows concordance and discordance among the 130 trans women with cis men partners on self-reported race/ethnicity, occupation, age, incarceration, and housing. Most African American/Black (72.7%) and White (64.3%) trans women reported having at least one cis man partner of the same race/ethnicity. No trans women who identified as Asian/Pacific Islander or Native American reported having a cis man partner of the same race/ethnicity. Slightly more than half of trans women in the age range of 40–49 years old reported having cis men partners from different age ranges (52.9%). Slightly more than half of trans women with a history of incarceration had cis men partners who also had a history of incarceration (55.4%). Almost half of trans women who currently lived in a single room occupancy (SRO) hotel had a sexual partner with the same living situation (43%), and of trans women who were currently homeless, 22% had a partner who was also currently homeless. Of 101 trans women with cis men partners who reported that they were not living with HIV, 11 had at least one partner who was living with HIV or their HIV status was unknown (10.9%). Of 28 trans women with cis men partners who reported that they were living with HIV, 57.1% had at least one partner who was not living with HIV or their partner’s status was unknown.

Most trans women who reported exchanging sex for drugs or money in the past 3 months were trans women with cis men sexual partners (97.3%, p = 0.02) (Table 4). Trans women with cis men partners had 8.04 times the odds of exchanging sex for drugs or money compared to trans women with trans women partners (p = 0.05, 95% CI: 1.04–62.01). This association remained statistically significant after adjusting for age and race (OR 8.45, p = 0.05, 95%CI: 1.04–68.75) (Table 5). Most trans women who reported having receptive condomless anal sex were trans women with cis men partners (92.6% vs. 7.4%, respectively). The odds of engaging in receptive condomless anal sex was 4.49 times higher among trans women with cis men partners compared to trans women with trans women partners (p < 0.05, 95%CI: 1.70–11.84). This association gained strength after adjusting for age and race (AOR 7.21, p = 0.02, 95%CI: 2.32–22.46). The odds of engaging in either insertive or receptive condomless anal sex while using substances was 3.43 times higher among trans women with cis men partners compared to trans women with trans women partners (p = 0.03, 95%CI: 1.09–10.76). All trans women who self-reported living with HIV were trans women with cis men partners (100%). This put their odds of living with HIV at 8.76 times higher than trans women without cis men partners (p = 0.01, 95%CI: 1.45-+inf).

## Discussion

This study fills important gaps in what we know about the context of structural disadvantage that amplifies HIV risk for trans women and their cis men sexual partners. Trans women with cis men partners and their cis men partners faced an extraordinary structural disadvantage. We found that significantly more trans women with cis partners engaged in sex work, pointing to income insecurity (15). Most trans women engage in sex work to meet needs for survival that require work in the informal economy due to gender-based discrimination (16, 17). Some also do sex work to meet gender-related surgery goals and for better pay that other jobs (16). Although sex work can be a source of income (18), it comes with threats of violence, criminalization, mental distress, and risk for HIV infection (16, 17, 19). Trans women who do sex work may be at risk of HIV for a variety of reasons, including the number of partners they have increasing chances of HIV exposure, inability to negotiate condom use, and sexual violence (20). We also found that although more than half of trans women with cis men partners had stable housing, 36% of them did not. Of trans women who single room occupancy hotels (SROs), 43% of their cis men partners had the same living situation. Living in an SRO has been associated with numerous health risks including substance use, mental illness, and HIV risk (21, 22), which cis men partners and trans women may face. For trans women, unstable housing is associated with poor HIV care outcomes (23) (24), and elevated risk behavior (25), as are incarceration and low income (26). Half of trans women with cis men partners who had a history of incarceration also had partners with a history of incarceration. Incarceration is associated with under-employment, as we observed in our study, which ultimately effects income and lifetime earnings leading to poverty for many people (27). A study conducted in Los Angeles with heterosexually identified cis men who occasionally had sex with trans women found similar levels of structural disadvantage as most had low income, high unstable housing, low education and more than 80% had been incarcerated (28). It has long been recognized that HIV is a pandemic of social disadvantage (29). These data situate trans women’s risk within their partnering with cis men who may face similar structural disadvantage, which may result in amplified risk for both partners.

We also found that trans women in partnerships with cis men were significantly more likely to report condomless receptive anal sex than trans women with trans women partners. An important contextual factor from this analysis is that almost all partners of trans women in our dataset were assigned male sex at birth, yet condomless receptive anal sex was more common among trans women with cis men sexual partners. Research has established that the primary mode of HIV risk for trans women is condomless receptive anal sex (5, 30, 31). Trans women with cis men partners were also significantly more likely to identify as heterosexual than trans women with trans women sexual partners, and sexual risk may be particularly prevalent in trans women’s sexual relationships with cis heterosexual partners. In a study of 80 trans women who have lived with HIV for anywhere from 4 to 34 years, they reported identifying as trans women when they acquired HIV and most acquired it from their heterosexual cis men partners (32). An online study of cis men with trans women sexual partners found that heterosexual cis men engaged in significantly more sexual risk behaviors compared to gay, bisexual, and queer cis men partners (4). Research with cis men living with HIV found they were less likely to use condoms with trans women than cis women or men sexual partners because they assumed trans women were already living with HIV. Policy and intervention approaches are needed that recognize that trans women most at risk of HIV are likely to identify as heterosexual, have cis men sexual partners, and face significant structural disadvantage.

Notably, there were no significant differences in recent HIV testing or PrEP among trans women with cis men partners compared to those without cis men partners. The fact that there was equal access to HIV prevention services for all partner types is positive and speaks to effectiveness in focused efforts in California to meet trans women’s HIV prevention needs. We also identified high sexual mixing by race, which may serve as a protective factor for trans women of color in partnerships with cis men as has been found among Black MSM (33).

## Conclusions

The primary limitation to this study was recruitment challenges we encountered from having to move the study from in person to remote due to Covid-19 shelter in place ordinances. This change limited our ability to obtain HIV biomarkers via rapid tests as was initially proposed and to rely on self-reported data. Despite these challenges, findings from this study fill important gaps in understanding trans women’s HIV risks within partnerships with cis men and the structural disadvantage cis men partners of trans women face. Research to improve impact in HIV prevention for trans women will need to incorporate interventions for their cis men sexual partners as well. Lastly, research shows that healthy behaviors and positive health outcomes are exhibited among trans women and their sexual partners who have full support of their relationships from society and those close to them, so interventions to address partnership dynamics may be most effective if acceptance is a key pilar (34, 35).

## Figures and Tables

**Table 1:** Demographic characteristics, social determinants, and HIV status of trans women participants (N = 156) and their sexual partners (N = 336)

	Trans women	Sexual partners
	N = 156 (100%)	N = 336 (100%)
Sex at birth^a^	156 (100)	321 (97.6)
Male	0 (0)	8 (2.4)
Female		
Gender identity^a^	0 (0)	262 (81.9)
Man	115 (73.7)	58 (18.1)
Transgender woman	36 (23.1)	0 (0)
Woman	4 (2.6)	0 (0)
Non-binary		
Sexual orientation^a^	73 (47.1)	185 (59.1)
Straight/heterosexual	7 (4.5)	18 (5.8)
Gay/lesbian	19 (12.3)	50 (16.0)
Bisexual	26 (16.8)	34 (10.9)
Pansexual	22 (14.2)	21 (6.7)
Queer	2 (1.3)	3 (1.0)
Questioning	6 (3.9)	2 (0.6)
Other		
Race/ethnicity	22 (14.1)	79 (23.7)
African American/Black	10 (6.4)	14 (4.2)
Asian/Pacific Islander	37 (23.7)	71 (21.3)
Latino/a/x	3 (1.9)	2 (0.6)
Native American	43 (27.6)	140 (42.0)
White	39 (25.0)	23 (6.9)
Multiracial/ethnic	2 (1.3)	4 (1.2)
Other		
Employment	50 (32.1)	40 (15.5)
Unemployed	75 (48.1)	169 (65.5)
Employed	10 (6.4)	11 (4.3)
Living on entitlements	21 (13.5)	38 (14.7)
Other		
Age group	54 (34.6)	111 (34.1)
18–29	49 (31.4)	117 (35.9)
30–39	21 (13.5)	55 (16.9)
40–49	32 (20.5)	43 (13.2)
50+		
Incarceration^a^	72 (46.8)	174 (68.5)
No	82 (53.3)	80 (31.5)
Yes		
Housing stability	102 (66.2)	226 (75.3)
Stable (rent/own)	52 (33.8)	74 (24.7)
Unstable (SRO, supportive housing, homeless/shelter/couch surfing or other)		
HIV status	124 (79.5)	262 (89.7)
Negative	31 (19.9)	19 (6.5)
Positive	1 (0.6)	11 (3.8)
Unknown		

Note:

a Answer don’t know, refuse, not applicable were coded as missing. Missing sex at birth for partners (N = 7), Missing gender identity for trans women (N = 1), missing gender identity for partners (N = 16), missing sexual orientation for trans women (N = 1), missing sexual orientation for partners (N = 23), missing race/ethnicity for partners (N = 3), missing employment for partners (N = 78), missing age for partners (N = 10), missing incarceration for trans women (N = 2), missing incarceration for partners (N = 82), missing living situation for trans women (N = 2), missing living situation for partners (N = 36), missing HIV status for partners (N = 44).

**Table 2:** Differences between trans women participants with cis gender and non-cis gender men partners (N = 152)

Characteristics	Trans women with cis men partners	Trans women with trans woman partners	P-value
	N = 130 (85.5%)	N = 22 (14.5%)	
Age	37.5 (12.2)	33.6 (7.7)	0.15
Mean (SD)	34.5	32	
Median			
Age group	47 (36.2)	7 (31.8)	0.07
18–29	35 (26.9)	11 (50)	
30–39	17 (13.1)	3 (13.6)	
40–49	31 (23.9)	1 (4.6)	
50+			
Gender identity^a^	26 (20.2)	8 (36.4)	0.12
Transgender woman	100 (77.5)	13 (59.1)	
Woman	3 (2.3)	1 (4.6)	
Non-binary			
Had vaginoplasty	14 (10.8)	4 (18.2)	0.30
Number of sexual partners, past 12 months	15.4 (53.1)	3.7 (3.7)	0.34
Mean (SD)			
Partner types, past 12 months^b^	63 (68.5)	15 (83.3)	0.26
Main	58 (63.0)	11 (61.1)	0.88
Casual	23 (25.0)	1 (5.6)	0.12
Exchange			
Sexual orientation^a^	71 (55.0)	0 (0)	0.00
Straight/heterosexual	3 (2.3)	4 (18.2)	
Gay/lesbian	17 (13.2)	1 (4.6)	
Bisexual	20 (15.5)	6 (27.3)	
Pansexual	14 (10.9)	7 (31.8)	
Queer	1 (0.8)	1 (4.6)	
Questioning	3 (2.3)	3 (13.6)	
Other			
Race/ethnicity^b^	22 (16.9)	0 (0)	0.00
African American/Black	8 (6.2)	2 (9.1)	
Asian/Pacific Islander	36 (27.7)	0 (0)	
Latino/a/x	3 (2.3)	0 (0)	
Native American	28 (21.5)	14 (63.6)	
White	32 (24.6)	6 (27.3)	
Multiracial/ethnic	1 (0.8)	0 (0)	
Other			
Education	94 (72.3)	11 (50.0)	0.036
Less than undergraduate degree	36 (27.7)	11 (50.0)	
Undergraduate degree or more			
Employment	59 (45.4)	16 (72.7)	0.018
Employed	71 (54.6)	6 (27.3)	
Not employed			
Had history of incarceration^a^	74 (57.8)	4 (18.2)	<0.001
Number of times incarcerated^a^	4.11 (7.5)	0.18 (0.4)	0.02
Mean (SD)			
Housing Stability	83 (63.9)	18 (81.8)	0.142
Stable	47 (36.2)	4 (18.2)	
Unstable			

Note:

a Answer don’t know, refuse, not applicable were coded as missing. Missing gender identity (N = 1), missing sexual orientation (N = 1), missing incarceration (N = 2).

b Multi-responses were allowed.

**Table 3:** Concordance in characteristics and social determinants among trans women with cis men sexual partners (N = 130)

	Concordance
Race/ethnicity	16 (72.7)
African American/Black	0 (0)
Asian/Pacific Islander	21 (58.3)
Latino/a/x	0 (0)
Native American	18 (64.3)
White	11 (34.4)
Multiracial/ethnic	0 (0)
Other	
Age	36.8 (12.6)
Mean (SD)	32
Median	
Age group	33 (70.2)
18–29	25 (71.4)
30–39	8 (47.1)
40–49	20 (64.5)
50+	
History of incarceration	46 (85.2)
No	41 (55.4)
Yes	
Current living situation	72 (86.8)
Rent/own	7 (43.8)
SRO	2 (16.7)
Supportive housing	2 (22.2)
Homeless/shelter/couch	2 (20)
Other	
Housing Stability	72 (86.8)
Stable	13 (15.3)
Unstable	
HIV Status	90 (89.1)
Negative	12 (42.9)
Positive	1 (100)
Unknown	

**Table 4:** HIV-related risk and protective factors by partnership type comparing trans women in cis men partnerships to trans women in non-cis partnerships (N = 152)

	Trans women with non-cis men partnerships	Trans women with cis men partnerships	P-value
	N = 22	N = 130	
Risk behaviors and HIV status			
Number of exchanged partners, past 12 months^a^	0.1 (0.3)	3.6 (11.8)	0.167
Exchanged sex for drugs or money, past 3 months^a^	1 (4.6)	36 (27.7)	0.016
Insertive condomless anal sex, past 12 months^a^	4 (18.2)	24 (18.5)	1.000
Receptive condomless anal sex, past 12 months^a^	7 (31.8)	88 (67.7)	0.001
Insertive or receptive condomless anal sex while using substances, past 12 months^a^	5 (22.7)	56 (43.1)	0.072
Had concurrent sex	21 (95.5)	99 (83.2)	0.197
Injected drugs, past 6 months^a^	0 (0)	8 (6.2)	0.603
Self-reported HIV positive	0 (0)	28 (21.5)	0.023
With the most recent partner who is HIV positive^b^	0 (0)	12 (10.7)	0.215
Protective behaviors			
Ever engaged in HIV care	--	28 (21.5)	--
Currently receive HIV care	--	26 (20.0)	--
Currently on ART	--	26 (20.0)	--
Ever on PrEP^a^	10 (45.5)	67 (51.5)	0.598
On PrEP, past 6 months^a^	5 (22.7)	44 (33.9)	0.338
Knew HIV status of the most recent partner	6 (28.6)	37 (33.0)	0.688
Ever been tested for HIV	22 (100)	129 (99.2)	1.000
Virally suppressed^a^	--	24 (98.4)	--

Note:

a Answers not applicable were coded as 0.

b Answers don’t know or refuse were coded as missing. Missing HIV positive status of the most recent partner (N = 18), missing know partner’s HIV status (N = 19).

**Table 5:** Odds ratios for HIV risk and protective behaviors of trans women with cis men partners compared to trans women with trans women partners (N = 152)

	OR	95% CI	AOR^a^	95% CI
Risk behaviors and HIV status				
Exchanged sex for drugs or money, past 3 months	8.04	1.04–62.01	8.45	1.04–68.75
Insertive condomless anal sex, past 12 months	1.01	0.32–3.28	1.68	0.47–6.06
Receptive condomless anal sex, past 12 months	4.49	1.70–11.84	7.21	2.32–22.46
Insertive or receptive condomless anal sex while using substances, past 12 months	2.57	0.90–7.40	3.43	1.09–10.76
Concurrent sexual partners	0.24	0.03–1.85	0.20	0.02–1.71
Self-reported HIV positive	8.76	1.45 - +inf		
Protective behaviors				
Ever on PrEP	1.28	0.52–3.16	1.31	0.48–3.55
On PrEP, past 6 months	1.74	0.60–5.03	1.51	0.48–4.79
Knew HIV status of the most recent partner	1.23	0.44–3.44	1.33	0.44–4.06

Note:

a Adjusted for age and race/ethnicity

## Data Availability

The data that support the findings of this study are available on request from the corresponding author. The data are not publicly available due to privacy or ethical restrictions.
